# OMICS in Fodder Crops: Applications, Challenges, and Prospects

**DOI:** 10.3390/cimb44110369

**Published:** 2022-11-03

**Authors:** Pawan Kumar, Jagmohan Singh, Gurleen Kaur, Paul Motunrayo Adunola, Anju Biswas, Sumandeep Bazzer, Harpreet Kaur, Ishveen Kaur, Harpreet Kaur, Karansher Singh Sandhu, Shailaja Vemula, Balwinder Kaur, Varsha Singh, Te Ming Tseng

**Affiliations:** 1 Agrotechnology Division, Council of Scientific and Industrial Research-Institute of Himalayan Bioresource Technology, Palampur 176061, India; 2 Department of Genetics and Plant Breeding, CCS Haryana Agricultural University, Hisar 125004, India; 3 Division of Plant Pathology, Indian Agricultural Research Institute, New Delhi 110012, India; 4 Krishi Vigyan Kendra, Guru Angad Dev Veterinary and Animal Science University, Barnala 148107, India; 5 Horticultural Sciences Department, University of Florida, Gainesville, FL 32611, USA; 6 Agronomy Department, University of Florida, Gainesville, FL 32611, USA; 7 Department of Agronomy, Horticulture, and Plant Science, South Dakota State University, Brookings, WA 57007, USA; 8 Department of Plant and Environmental Sciences, New Mexico State University, Las Cruces, NM 88001, USA; 9 Department of Biological Sciences, Auburn University, Auburn, AL 36849, USA; 10 Department of Agricultural and Environmental Sciences, Tennessee State University, Nashville, TN 37209, USA; 11 Department of Crop and Soil Sciences, Washington State University, Pullman, WA 99163, USA; 12 Agronomy Department, UF/IFAS Research and Education Center, Belle Glade, FL 33430, USA; 13 Department of Entomology, UF/IFAS Research and Education Center, Belle Glade, FL 33430, USA; 14 Department of Plant and Soil Sciences, Mississippi State University, Starkville, MS 39759, USA

**Keywords:** alfalfa, cowpea, genomics, maize, metabolomics, oats, phenomics, proteomics, sorghum, transcriptomics

## Abstract

Biomass yield and quality are the primary targets in forage crop improvement programs worldwide. Low-quality fodder reduces the quality of dairy products and affects cattle’s health. In multipurpose crops, such as maize, sorghum, cowpea, alfalfa, and oat, a plethora of morphological and biochemical/nutritional quality studies have been conducted. However, the overall growth in fodder quality improvement is not on par with cereals or major food crops. The use of advanced technologies, such as multi-omics, has increased crop improvement programs manyfold. Traits such as stay-green, the number of tillers per plant, total biomass, and tolerance to biotic and/or abiotic stresses can be targeted in fodder crop improvement programs. Omic technologies, namely genomics, transcriptomics, proteomics, metabolomics, and phenomics, provide an efficient way to develop better cultivars. There is an abundance of scope for fodder quality improvement by improving the forage nutrition quality, edible quality, and digestibility. The present review includes a brief description of the established omics technologies for five major fodder crops, i.e., sorghum, cowpea, maize, oats, and alfalfa. Additionally, current improvements and future perspectives have been highlighted.

## 1. Introduction

Fodder, also known as hay, silage, or forage, is any crop or crop by-product used as feed for livestock, making it an essential source of protein and fat [1]. In crop production, fodder is the end product, the quality of which significantly affects the livestock. The by-products of crops, including cereals, legumes, grasses, and other crops, contribute considerably to fodder. Continuous improvement in animal breeds requires high-quality fodder to feed them. 

Biomass yield is the primary target in forage crop improvement programs worldwide. Cultivars or varieties with rapid growth and regrowth are targeted in breeding programs. Therefore, traits such as heading date, flowering time regulation [2,3,4], and delayed senescence [5] have been targeted for increasing biomass yields. Throughout the world, most fodder (up to 70%) is produced on cultivable land, and the major problem in silage production is the lack of protein in fodder [6]. The increasing population of humans and cattle will create and increase competition in future food and forage production and affect natural resources, particularly land and water [7]. 

**Figure 1:**
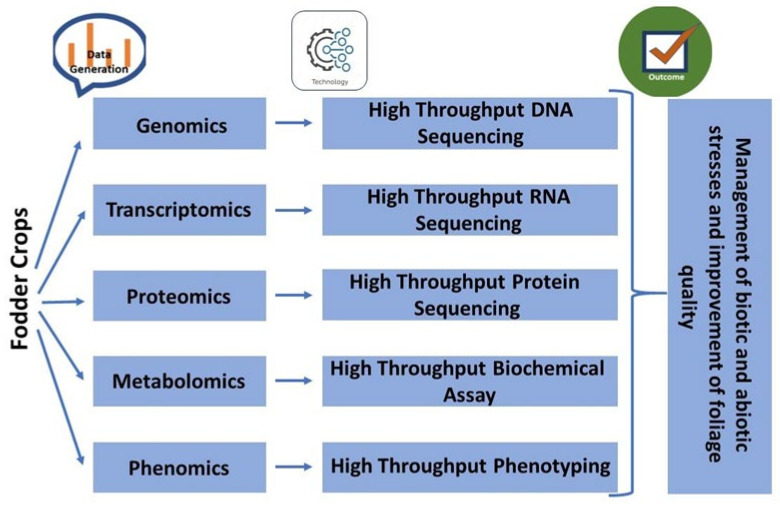
Multiple omics approach for fodder quality improvement in crop plants.

With the increasing challenges in agriculture, we also have great opportunities, especially with booming advances in omic technologies. Advancements in biotechnology and computational sciences have made it feasible to generate omics data for large sets of plants, varieties, or species at a reasonable price [8]. Handling and analyzing large and complex omics data has become possible with the availability of advanced computation and bioinformatics tools. The use of different omics led to the identification of genes, their functions, type of RNA or protein involved, their structure, and pathway involved in the appearance of the final morphological character [9] (Figure 1). Comparative omic analysis of different regions of varying environmental conditions makes it possible to identify genes essential for adaptation [10]. Identified genes can be manipulated or transferred to develop new hybrids or varieties with desirable characteristics. 

**Figure 2:**
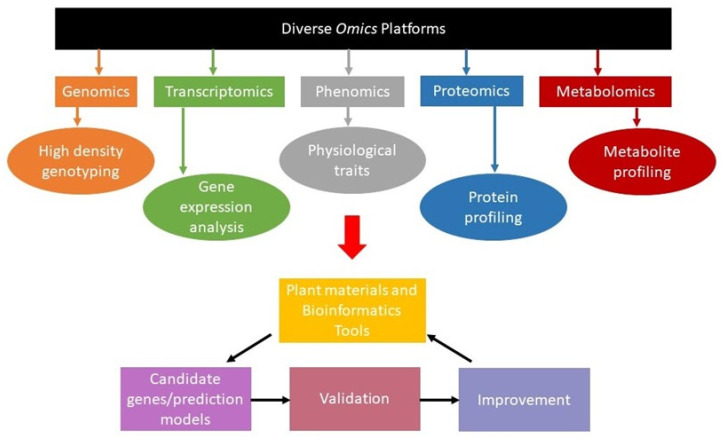
Graphical representation of the application of different omics approaches and their use in crop improvement.

**Figure 3:**
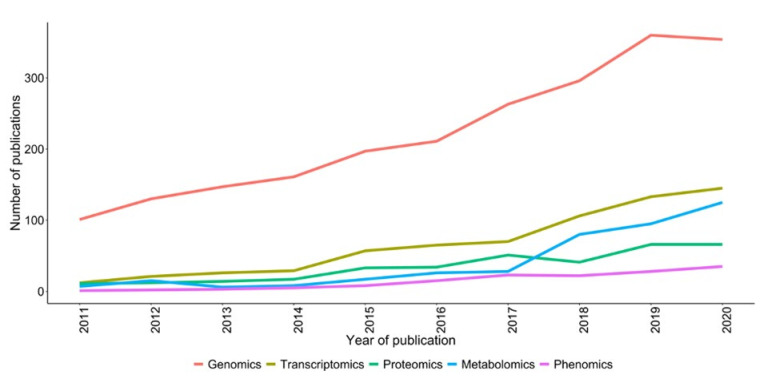
Graphical presentation showing the trend of publications mentioning omic approaches to improve fodder crops in the last decade. The search was made using the associated omics technique and the crop name keyword in the abstract. Source: PubMed, dated 5 August 2022.

The integration of multi-omics has been successfully performed for yield increment and biotic and abiotic stress tolerance/resistance development in agricultural crops (Figure 2). However, in fodder crops, in the recent past, a handful of studies have been conducted in crop plants using molecular biology approaches of different omic technologies, including genomics, transcriptomics, proteomics, metabolomics, and phenomics [11,12,13,14,15,16], to achieve higher yields along with quality fodder (Figure 3).

Genomics aims to sequence, characterize, and study the genetic compositions, structures, organizations, functions, and networks of an entire plant genome [17]. Genomics approaches are beneficial when dealing with complex traits, as these traits usually have a multi-genic nature and a significant environmental influence [18]. Advances in plant genomics have given new methodologies for plant breeding, which have improved and accelerated the breeding process in many ways (e.g., association mapping, marker-assisted selection, ‘breeding by design,’ gene pyramiding, genomic selection, etc.) [19,20,21]. Transcriptomics utilizes high-throughput sequencing platforms to generate transcript data from RNA sequencing, microarray, and serial analysis of gene expression (SAGE) to elucidate non-coding and coding RNA expression profiles to plant biotic and abiotic stresses [22].

Proteomics is the comprehensive study of all proteins in a complex biological system (plants, animals, and humans) at a particular time snapshot [23]. Proteomic analysis is required to estimate the abundance of different proteins, changes observed due to various post-translational modifications, their subsequent function, and localization [24]. It provides a snapshot of different metabolic processes, their consequent interactions, and their effects on other regulatory pathways of biological processes. Hence, a proteomic study is essential to decipher different reactions of pathways at different stresses and times [25].

Metabolomics is an advanced biotechnique that identifies functionally active metabolites, their roles, and the diverse biochemical processes that the metabolites play in plant genotypes and phenotypic expressions [26]. It is commonly applied to explore different aspects of plant breeding, the regulatory mechanisms related to plant growth and development (such as those related to crop productivity and performance), adaptation to biotic and abiotic stresses, nutritional improvement, and the selection of cultivars for agriculture. Metabolomes are simply metabolites (both secondary and primary) having a low molecular weight (usually <1500 Da), including their precursors and intermediates, of the corresponding biosynthetic pathways. Understanding the plant metabolomic processes would be beneficial for improving crop yield and human nutritional aspects in crop-breeding programs [27]. Based on the purpose of the study, metabolomics can be differentiated into two types, targeted and untargeted. Targeted metabolomics deals with the absolute quantification of one or a few metabolites in a set of predefined known substances. Therefore, the targeted approach tends to be highly sensitive and quantitative and can help discover the metabolites associated with specific stress. Targeted metabolomics measures the relative abundances of several hundred to thousands of all detectable metabolites. The untargeted approach, on the contrary, measures mass spectrometric features of unknown metabolites and thus enhances the chances of sensing unintended effects.

All genomics techniques (linkage and association mapping, genome-wide association studies, marker-assisted selection, genome-assisted selection, haplotype-based breeding, etc.) require accurate phenotyping information for selecting a breeding program [27]. Collecting accurate phenotyping information is challenging in large-scale breeding programs, particularly those that screen thousands of genotypes per year at multiple locations. The condition is further worsened for the fodder crops where multi-cuts are performed every season [16]. Recent developments in phenomics have lagged behind genomics and transcriptomics approaches; however, the last decade (2010–2019) witnessed the growth of various phenomics tools for deployment under field and controlled conditions [28]. These phenomic tools have shown the potential to phenotype biotic and abiotic stress and agronomic and physiological traits in fodder crops [29,30]. Several aerial and ground-based platforms carrying various imaging sensors and cameras have been used to accurately, precisely, and rapidly measure traits at multiple stages [31]. More information about these sensors, imagers, platforms, and data analysis methods used for fodder crops is provided in the phenomics section below. The current review discusses the progress in maize, sorghum, oat, alfalfa, and cowpea crops concerning their fodder using various omics, bottlenecks, and future prospects.

### 1.1. Sorghum

Sorghum (*Sorghum bicolor* L.) is a multipurpose crop used around the world as fodder, feed for poultry, grain for human consumption, and an essential source of biofuel production [32]. Sorghum is resistant to drought and waterlogging and is grown in different soil conditions. Being a water stress-tolerant crop, it can be cultivated in semi-arid areas with low precipitation. Its origin is marked in Africa; being the center of origin, it sustains the highest genetic diversity in sorghum genotypes. The vast diversity of sorghum germplasm is maintained at various centers, such as ~40,000 accessions at the USDA-ARS-National Plant Germplasm System, ~38,000 at ICRISAT, and >16,000 at the National Crop Gene Bank of China [33]. Sorghum (2*n* = 20) is a potential model plant for C4 type plant studies due to its small genome size as compared to other C4 plants such as sugarcane (10 Gbp) and maize (2.5 Gbp) [34]. The first whole-genome sequence of sweet sorghum was generated through the long-read sequencing platform of pacific biosciences [35]. 

The GWAS studies based on 245 accessions genotyped by 85,585 SNPs and phenotyped under four different environments identified 42 SNPs related to fodder quality traits (crude protein, acid detergent fiber, neutral detergent fiber, cellulose, and hemicellulose) by using mrMLM software [14]. The GWAS panel with 265,487 SNPs is used to detect the inflorescence and plant height traits [36,37], heat and cold stress [38], and disease resistance for anthracnose [39], stalk rot [40], and gray mold disease [41]. The biomass accumulation in the stem is essential in fodder sorghum, which marks the importance of cell wall biosynthesis. The GWAS analysis by the hidden Markov model elucidated 520 genes belonging to 20 gene families associated with cell wall polymers such as pectin, cellulose, and hemicelluloses [42]. The sweet sorghum, with better sugar accumulation in its stem, provides good succulent fodder for animals. The GWAS and QTL mapping identified various QTLs associated with sugar accumulation. The GWAS study reveals the high stem juiciness, juice volume, and sugar yield as compared to grain sorghum cultivars, but with no significant variation in Brix value (sugar concentration) [43]. A QTL responsible for crude protein, acid detergent fiber, and neutral detergent fiber was elucidated [14]. The four QTLs regulating plant height by determining internode length were reported as *dw1, dw2, dw3,* and *dw4* [44] The *dw1* (membrane protein) and *dw2* (protein kinase) are extensively used in sorghum breeding programs [45,46]. The brown mid-rib trait is characterized by low lignin content, and better biomass is of great importance for fodder sorghum. The four alleles, *bmr2*, *bmr6*, *bmr12*, and *bmr19*, governed the brown mid-rib traits, and the bmr12 class reduced lignin and enhanced digestion efficiency [47]. The tb1 gene, a transcription factor influenced by phytochrome B, is involved in sorghum tillering [48]. In another study, the heritability of forage traits such as plant height, leaf length, leaf width, dry weight, number of leaves, green fodder, and dry weight was studied, and the maximum heritability of dry weight was 98.6%, followed by 77.64% for leaf width. Leaf width has a positive correlation and leaf length has a negative correlation with the number of leaves, dry weight, and green fodder [49]. Another important trait of sorghum, i.e., the stay-green feature, was investigated in different sorghum genotypes to determine which genes contribute to this feature. This study identified four main QTLs, Stg1–Stg4, that contribute to the stay-green property in sorghum. Identifying these QTLs is a foundation for further research into stay-green physiology, QTL interaction, and map-based cloning of the genes that drive the stay-green phenotype [50].

The transcriptomic analysis of two drought-challenged sorghum cultivars from plant emergence to post-anthesis, with 400 transcriptomes, revealed the temporal transition in gene expression patterns in leaves and root tissue. The modulation in drought pathways, the difference in transcript photosynthesis, and relative oxygen species indicate the drought tolerance mechanism in sorghum plants to survive under water stress [51]. The dynamics of root architecture concerning apoplastic barriers, such as casparian strips and suberin lamellae, which help survival in barren saline soil, were elucidated by transcriptome analysis of distinct development stages of roots [52]. The transcriptomics studies revealed that sweet sorghum with high sugar content had a better growth rate and showed better biomass product efficiency under abiotic stress such as salinity and water lodging [53]. Another expression study of sweet sorghum (KIT1) and grain sorghum (Razinieh) elucidated that the expression of sugar transporters such as SbSUT1, SbSUT2, and SbSUT6 is higher in the sweet genotypes, and their expression is significantly enhanced when subjected to saline conditions [54]. The transcriptome analysis of grain sorghum, sweet sorghum, and a cross between the two (as line R9188) revealed a specific carbon allocation trend during sugar accumulation in its stem. The line R9188 had starch metabolism and sucrose level, whereas its parent seed sorghum had fully activated sucrose and starch metabolism with high sugar concentration [43]. 

Proteomic investigation helps identify potential markers among genotypes that can be utilized in breeding programs. Although the sorghum genome sequence was published five years ago, proteomics of sorghum under drought and salt stress is still in its early stages, and there is limited research [55]. Drought tolerance is influenced by stress perception, signal transduction pathways, and changes in gene expression, all of which affect plant physiology and metabolism [56]. Pre- and post-flowering drought stress in sorghum has drastically reduced grain yield. In a study, post-flowering drought stress in sorghum leaf tissue was examined using two-dimensional gel electrophoresis and matrix-assisted laser desorption ionization-time of flight mass spectrometry (MALDI-TOF-MS). This study concludes that the plant increases the activity of key proteins as a defensive mechanism in the sorghum plant to overcome drought stress [57]. Protein expression patterns and physiological analysis of sorghum root to Polyethylene Glycol (PEG)-induced drought stress at the seedling stage. This study achieved protein identification using Coomassie Brilliant Blue-stained 2-DE gels. Out of sixty-five proteins identified, the levels of 43 protein spots increased, and 22 decreased in drought conditions. These proteins are involved in molecular mechanisms such as protein synthesis and carbohydrate and energy metabolism, contributing to drought tolerance in sorghum [10]. In a study, drought-sensitive (ICSB338) and enhanced drought-tolerant (SA1441) sorghum were compared using proteomic techniques. This study showed that drought-tolerant sorghum plants preserve leaf water content through the stomatal shutdown, making them survive under drought-stress conditions compared to drought-sensitive plants [58]. 

Metabolomic approaches have revealed that drought-stressed plants accumulate a variety of metabolites such as amino acids, organic acids, polyamines, and lipids to protect plant cells against oxidative stresses. In a study by Rajarajan et al. [59], the relative changes of metabolites, such as total carbohydrates, amides, and lipids, were investigated in two sorghum genotypes. The results further revealed a change in these metabolites in the genotypes under drought stress, i.e., DRT1019 had a higher absorbance value than the ICSV95022 genotype. Such alterations in the levels of metabolites in response to drought stress possibly play critical roles in adjusting the cellular metabolism of water-stressed plants. The results of this study provide promising candidate genes for drought tolerance in sorghum that can be used as potential markers for drought tolerance breeding programs. Another recent study on sorghum cultivars highlighted the use of metabolomics to investigate the underlying biochemistry behind changes in seed color [60]. In the past, metabolomics has proven crucial for studying plant × environment interactions and has been applied in the metabolomics-assisted breeding of crops. The metabolomics-based investigation reports on the characterization of multi-parametric metabolic re-programming that underlies the induced defense mechanisms in sorghum plants responding to *Colletotrichum sublineolum* infection. Tugizimana et al. [61] used an LC-MS-untargeted metabolomics approach supported by gene expression analyses, which was aimed at obtaining a comprehensive understanding of the defensive metabolism of sorghum in response to *C. sublineolum* inoculation. Multivariate data analysis identified 72 discriminatory/signatory biomarkers and 23 potential metabolic pathways, with nine being the most significant pathways and collectively defining the metabolic state of the induced resistance in sorghum. The hydrogen cyanide (HCN) in fodder sorghum is an essential trait as it is toxic to animals. The dhurrin [(S)-4-Hydroxymandelnitrile-D-glucopyranoside] in sorghum is metabolized to synthesize HCN. The correlation in transcriptome and metabolome studies undermined the 14 candidate genes of dhurrin metabolism. The p-hydroxybenzaldehyde (pHB) was highest at the early growth stage and reduced with maturity. The candidate genes also showed an expression peak at the seedling stage and decreased at the adult stage [62]. The metabolic profile and expression of dhurrin metabolism genes in sorghum provide the criteria for selecting accession for fodder breeding programs to prevent HCN toxicity in livestock or promote drought tolerance or pathogen resistance. 

Furthermore, phenotyping for abiotic stress and biomass accumulation is a tedious and expensive task, but the advent of phenotyping platforms and imaging sensors has opened various new avenues. To maintain stomatal conductance, sorghum can keep its stomata open under different turgor pressures. Canopy temperature depression is a surrogate for stomatal tolerance to select genotypes with better drought tolerance. It has been observed that canopy temperature (CT), water use efficiency, and yield have a high correlation in sorghum, which is possible using several thermal and multispectral imaging techniques [27]. Aerial phenotyping platforms, namely Unmanned Aerial Vehicles (UAVs), satellite imagery, and manned aircraft, have been used to measure biomass, CT, plant height, nitrogen and chlorophyll content, and biotic stresses in sorghum. UAVs are preferred over manned aircraft and satellites due to their high spatial and temporal resolution, low operation and hardware costs, and flexibility in operation. Watanabe et al. [63] used the red, green, and blue (RGB) and near-infrared (NIR) cameras installed on UAVs to measure plant height in sorghum, further deciphering the potential of remote sensing. Moderate to high correlations were observed in manual plant height notes and height data provided by the UAV. The use of robots is still not behind in sorghum, [64] used a low-cost and tracked mobile robot to collect plant height and stem width from each plot using stereo and depth sensors with very low error rates. For bioenergy sorghum, stem characteristics such as standability and juice yield are the key traits; however, their phenotyping is low throughput. Gomez and coworkers [65] developed medical X-ray computed tomography (X-CT) to image morpho-anatomical properties in sorghum to characterize genotypes based on pithiness ratio, stem diameter, and length. The growth angle for nodal roots influences the spatial distribution of mature plants and aids in drought adaptation. Joshi and a group of researchers [66] used the digital images collected in the root chambers using openGelPhoto.tcl software to reveal genetic variation for this trait. Several automated phenotyping platforms are coupled with computational systems to perform data analysis using dimensional reduction, classification algorithms, and a fusion of deep learning models. Most of the studies for sorghum phenotyping are either under controlled conditions or at a small scale; however, in the coming years, we will see the deployment of such tools and methods at a large scale for sorghum breeding.

### 1.2. Cowpea

Cowpea [*Vigna unguiculata* (L.) Walp.] is an herbaceous annual diploid crop (2*n* = 22) belonging to the Fabaceae family with a genome size of ~620 million base pairs [67]. It is mainly grown in the semi-arid tropics in Latin America, Africa, and South Asia [68]. It is a dual-purpose crop as it is an essential contributor to human food and livestock fodder. Being drought-tolerant, cowpea can be grown in semi-arid regions where other legumes do not grow well. It can also grow well in poor soils high in sand content with low organic and phosphorus contents [69,70]. The cowpea is mainly produced in Africa (80%) and some other parts of the world, including Asia, Brazil, and the United States, and all have substantial production [71]. Like other grain legumes, cowpea is a vital food crop in tropical and subtropical countries [72] because of its use mainly as a grain crop, a vegetable, or animal fodder. Although it occupies a smaller proportion of the crop area than cereals, cowpea contributes significantly to household food security in West and Central Africa [73], biotic-abiotic stress [74,75], seed harvests [76], nutrient continent [77], and physiological architecture [78]. Using cowpea as fodder is appealing in mixed crop/livestock practices. Both grain and fodder can be harvested from the same crop. It can also be used as an intercrop with maize, sorghum, sugarcane, cotton, and other crops [79]. It enhances the soil by recycling nutrients by fixing nitrogen with nodulating microbes. Cowpea is mainly grown for grains, it is rich in protein, ranging from 20 to 25% dry weight. Dried cowpea leaves, stems, and pod walls (known as haulm) can provide additional income for farmers as a feed resource. Cowpea fodder contains up to 18.6 g of protein per 100 g of dry weight. With all the benefits, cowpea remains a relevant crop for human and livestock nutrition, food security, and income for subsistence farmers [68]. The improvement of cowpea varieties was initially led by the International Institute of Tropical Agriculture (IITA), Nigeria, which developed and distributed improved cowpea varieties. Initially, the variety improvement at IITA focused on grain cowpea cultivars only [80]. With cowpea’s importance as a fodder crop, a systematic breeding program was initiated at the IITA to develop dual-purpose cowpea varieties that focused on combining high yield for both, i.e., grain and fodder, and resistance to biotic and abiotic stresses [68]. As cowpea is a highly self-pollinating crop, improved varieties have been developed using pure line selection, mass selection, pedigree breeding, single-seed descent, and backcrossing methods [81]. Recent studies showed that grain and fodder yield in cowpea are positively correlated, so improving the varieties for both traits is possible. However, there is no relationship between grain yield and fodder quality traits [82]. 

The genomic efforts for cowpea have been more recent and focused on molecular diversity and genetic linkage mapping [81]. Several marker systems such as allozymes, restriction fragment length polymorphism (RFLP) [83], amplified fragment length polymorphisms (AFLP) [84], DNA amplification fingerprinting (DAF) [85], random amplified polymorphic DNA (RAPD) [86,87], simple sequence repeats (SSRs) [88], cross-species SSRs from Medicago [89], inter-simple sequence repeats [90], sequence-tagged microsatellite sites (STMS) [91], and single nucleotide polymorphism (SNP) markers [91,92] have been used to study origins, domestication, and genetic variation in cowpea. A set of more than 1,500 single nucleotide polymorphism (SNP) markers on 442 cowpea landraces identified two major gene pools in cultivated cowpea in Africa [91,92]. Genotyping by sequencing (GBS) was applied to identify cowpea SNPs and estimate genetic diversity, population structure, and phylogenetic relationships. Another diverse set of 768 cultivated cowpea genotypes from 58 countries was studied using GBS SNP markers that revealed three gene pools (America, Africa, and Central West Asia) [91,93]. Lastly, a set of 368 cultivated cowpeas genotyped with 51,128 SNPs revealed six major subpopulations [94]. The genetic research on cowpeas was initiated with the development of the first linkage map for cowpeas using a population of 58 F2 plants from a cross between IT84S-2246-4 and TVNu 1963 [83]. A second cowpea genetic linkage map was developed using 94 F8 recombinant inbred lines (RILs) made from a cross between two cultivated cowpea genotypes, IT84S-2049 and 524B [95]. A third genetic map was made using 94 F8 RILs derived from a cross between a cultivated cowpea line, IT84S-2246-4, and a wild relative (*V. unguiculata* spp. *dekindtiana* var. *pubescens*) TVNu 110-3A [96]. After the development of an Illumina GoldenGate Assay, an SNP consensus map was developed with 928 SNP markers covering a total genetic distance of 680 cM was established based on the genotyping of 741 members of six bi-parental RIL populations derived from the following crosses: 524B × IT84S-2049, CB27 × 24-125B-1, CB46 × IT93K-503-1, Dan Ila × TVu-7778, TVu-14676 × IT84S-2246-4, and Yacine × 58–77 [97]. The resolution of this consensus genetic map was improved by genotyping 579 individuals from additional populations consisting of five RILs (from UCR–US, IITA–Nigeria, ISRA–Senegal, ZAAS–China) and two F4 populations [98]. After the availability of linkage maps, opportunities became available for QTL resolution, map-based cloning, genetic diversity, association mapping, and marker-assisted breeding. Synteny has been reported between cowpea and mung bean [99] based on RFLP-derived separate maps of both crops. Lucas et al. [98] also reported that 941 of 1107 total SNP markers, i.e., 85% mapped in cowpea, show homologs with soybean (*Glycine max*). The markers also showed synteny and collinearity in the soybean genome. Advancements in linkage maps led to the identification of QTLs for several desirable traits in cowpeas, including leaf shape, disease and insect resistance, maturity time, seed-related traits, flowering time, and pod-length variation [81,97,100]. With better comprehension of the genomic basis of variation, genome-wide association studies (GWAS) studies have been highlighted on the subjects of cowpea pod length [100], root architecture [101], cowpea plant improvement traits, as well as the flowering period [94]. These findings are appreciated because cowpea genetic diversity assessment is necessary for strengthening breeding programs to develop high-yielding dual-purpose cultivars with good grain and fodder yields [102]. It has been found that cowpeas have 85% macrosynteny with *Glycine max* and 82% with *Medicago truncatula*, which can help in comparative analysis to identify genomic regions for fodder yield and quality [103]. Paudel and coworkers (104) reported candidate genes for flowering time in cowpeas based on GWAS and showed high H2 estimates (0.72–0.95) for flowering time in cowpeas. Extensive collections of diverse cowpea accessions are conserved in the International Institute of Tropical Agriculture (IITA) (∼15,000 accessions), the United States Department of Agriculture–Genetic Resources Information Network (USDA-GRIN) (7737 accessions), and the University of California, Riverside, CA, United States (∼6000 accessions) [104]. Due to resource limitations in characterizing the whole collection, many conserved accessions in gene banks preclude their direct utilization in a breeding program. Therefore, a mini-core collection consisting of 298 lines from the IITA collection was genotyped based on GBS using 2276 SNP markers to make the germplasm’s characterization and utilization more practical [105]. Similarly, another mini-core collection, the University of California-Riverside Minicore (UCR Minicore), consisting of 368 accessions that included landraces and breeding materials from 50 countries, was also developed [94] and genotyped using a publicly available Cowpea iSelect Consortium Array [106]. This array consists of 51,128 assays developed from sequencing 36 diverse accessions and was released to facilitate easy, high-throughput genotyping in cowpeas [106]. A few reports of marker-assisted breeding in cowpea improvement, such as marker-assisted backcross, were used to transfer the Striga resistance gene from the breeding line IT93K-693-2 to three farmers’ preferred varieties, IT90K-372-1-2, KVx30-309-6G, and TN5-78 [107]. Three significant QTLs for bacterial blight, one on Vu09 (*qtlblb-1*) and two on Vu04 (*qtlblb-2* and *qtlblb-3*), which were responsible for 30.58%, 10.77%, and 10.63% of phenotypic variations, respectively, have been identified [108]. The QTL on Vu09 was introduced from cultivar V-16 into the bacterial leaf blight susceptible variety C-152 through marker-assisted backcrossing (MABC) [109].

The characterization of different parts of the cowpea plant through transcriptomics has been carried out in studies that express the diverse genes essential for cowpea growth and development. The stress-resilient genes have also been characterized, and their role in the overall improvement of cowpea has also been highlighted [110,111,112,113]. There has been minimal use of transcriptomics for cowpeas until now for fodder purposes. Genes for cowpea growth, development, and stress-related genes have been characterized using transcriptomics, which has a role in seed and pod development [110]. Marker-assisted breeding was used to introgress large seed haplotypes into a CB27 background with 22 g/100 seeds using a rare haplotype with large seeds at the Css-1 locus from the cowpea variety IT82E-18 (18.5 g/100 seeds) [114]. During two cycles of backcrossing based on genome-wide SNP markers, foreground and background selections resulted in families with very large seeds (28–35 g/100 seeds). Parasitic weeds, *Striga gesnerioides* (present in the dry savannah areas of West and Central Africa) and *Alectra vogelii* (found in eastern and southern Africa) can cause yield reductions of 73 to 100% [110,115]. One Striga plant can produce up to 90,000 seeds, which can be viable in the soil for 15 to 20 years [116]. There are seven races of Striga identified [117]. Race 1, 2, 3, 4, 5, 6, and 7 were found in Burkina Faso, Mali, Nigeria, and Niger, the Republic of Benin, Cameroon, Senegal, and Zakpota in the Republic of Benin, respectively [118]. Three Striga resistance genes, *Rsg-1*, *Rsg-2*, and *Rsg-3*, were identified in two cowpea lines [119]. Two duplicate genes, *Rav-1* and *Rav-2*, control cowpea resistance to Alectra [120]. Genes conferring resistance to Striga and Alectra were not found to be allelic or linked [121]. Aphid (*Aphis craccivora* Koch) is the first primary insect pest that affects cowpea growth early. It damages the seedlings in drought conditions [122]. A dominant gene (*Rac*) was identified in a germplasm line (TVu-3000) for resistance to aphids [123]. However, this gene became ineffective for aphid resistance. Then a cowpea wild relative, TVNu-1158, was found resistant to aphids in the seedling stage [124]. A set of RILs was developed using this wild relative as a parent, and some of the resistant RILs are now being used as parents in breeding programs to transfer resistance to aphids [125]. Recently, three cultivated cowpea accessions, TVu-6464, TVu-1583, and TVu-15445, with varying resistance levels to *A. craccivora* comparable to the level already found in an existing resistant TVu-801, were reported [126]. All these new sources of resistance could be used in pyramiding to develop new aphid-resistant cowpea varieties. The resistance mechanism in these three accessions was linked to low sucrose levels and high levels of kaempferol and quercetin (aglycones of phenolic compounds) [126]. The development and application of genomic tools for cowpea improvement is limited, and only a few relevant studies have been reported. A little progress has been recorded after identifying molecular markers associated with some desirable traits in the crop, but marker application in variety development is still minimal.

After decades of study on cowpea, many omics datasets are now accessible, which can be used to better understand the genetic relationship between *Vigna unguiculata* ssp. *unguiculata* and other species in the same genus, and their genetic variation. Furthermore, the availability of a genetic map and a genetic transformation protocol increased the opportunity to identify more genes and study their expression [127]. Various salt-tolerant cowpea cultivars have different protein profiles and use other techniques to deal with salt stress. Differential proteome responses in two cowpea cultivars to salt stress have been studied. LC–ESI–MS/MS revealed 22 differentially regulated proteins by salt and recovery. This study speculates that tolerance may be associated with maintaining an optimal enzymatic-protein level required for active energy metabolism, such as photosynthesis in salinity or recovery [128]. A similar study was conducted in cowpea to understand the mechanisms involved in drought-tolerant and drought-sensitive genotypes. One hundred eight differentially expressed proteins were identified using 2D E and MS that may be associated with drought response in both genotypes. Glutamine synthetase, CPN60-2 chaperonin, malate dehydrogenase, heat-shock proteins, and rubisco were identified as drought stress-response peptides expressed differentially in both genotypes. This study concluded that most of the proteins identified were related to photosynthesis, the critical mechanism for plant survival [115]. Protein expression patterns associated with embryogenic cell suspension prepared from friable embryogenic calli (FEC) were investigated in cowpea using two-dimensional gel electrophoresis protein mapping and mass spectrometry analysis. A total of 128 protein spots have been identified, including PR-4 (chitinase) and PR-10 (putative ribonuclease), as significant proteins playing a major role in the differentiation of pro-embryogenic masses into somatic embryos and defense against biotic and abiotic stresses in cowpeas [129].

Metabolomics tools can be deployed in identifying and monitoring physiological responses in plants and the metabolic pathways or linkages arising from the biotic and abiotic stress exerted upon plants. A study on the drought response of three cowpea landraces using leaf physiological and metabolite profiling assessment [130] used gas chromatography time of flight mass spectrometry (GC-TOF-MS) and reported that cowpea landrace A116 genotype performed best with the accumulation of 14 bioactive metabolites including proline, valine, rhamnose, raffinose, isoleucine, fucose, urea, alanine, sucrose, and putrescine. Several metabolites such as galactinol, proline, quercetin, rhamnose, and raffinose involved in drought tolerance have been identified using metabolomics tools in cowpeas [130,131]. Another study on metabolites (polyphenols and carotenoids) in *V. unguiculata* sprouts identified and quantitated 39 hydrophilic compounds using high-performance liquid chromatography (HPLC), electrospray ionization-mass spectrometry (ESI-MS), gas chromatography-mass spectrometry (GC–MS), and gas chromatography [132]. This study provides a new approach for enhancing the carotenoid and phenylpropanoid production of *V. unguiculata*. This metabolite profiling approach has been utilized to understand the molecular response of cowpea plants to abiotic stress and will have an enormous impact on crop yields. Similarly, advances in metabolomics could assist in identifying the various metabolites produced in response to heat stress and thus determine the complex signaling networks contributing to heat stress tolerance in cowpea [133]. Therefore, metabolomics could assist in screening cowpea lines for heat tolerance, but such studies are mainly lacking in cowpeas.

Phenotyping is crucial to work on any trait development. High throughput phenotyping can be a game-changer in plant breeding. Biotic-abiotic stress, seed, plant vigor, nutrition, and flowering time are some of the traits of interest of cowpea to improve for either food or fodder purposes. Despite the importance of phenomics, a minimal amount of work has been conducted on phenomics for this crop [74,75,76,77,78,134]. So, we think phenomic-assisted selection will be prospective for this specific crop. 

### 1.3. Maize

Maize (*Zea maize*) is a multi-purpose cereal crop grown for food, feed, and industrial purposes [135,136,137]. About a billion people worldwide consume maize as a staple food [138]. Globally, it is one of the most important cereal crops, and it is adaptable to grow in different environmental conditions, generating billions of dollars in revenue annually [139,140,141]. Maize has evolved into one of the most essential model crops for understanding the genetics and genomes of plants [142,143,144]. The complexity of the diverse maize genome has kept researchers interested in this crop studying cytogenetics and genomics. Aside from rice and wheat, maize is the most studied food crop for the elucidation of cells and tissue molecular composition to reveal the functional machinery of the plant genome. There are approximately 30,000 to 40,000 genes in a maize genome of 2.4 billion base pairs. The variation available throughout the world in the maize crop has allowed plant breeders and geneticists to improve this crop continuously.

Birchler [145] constructed an early genetic map for maize on stained chromosomes based on the cytogenetic position of the relative gene. With the development of molecular [146,147,148,149] and sequencing technology [150,151], several attempts have been made for maize genome sequencing. Among all those, B73 RefGen_v4 [152] is the most accurate assembly of maize to date. It was based on PacBio sequencing and high-resolution optical mapping. To separate the genetic architecture of complex traits, linkage mapping and association mapping are the most widely used approaches in maize and other crops. Linkage mapping is a conventional approach and has its limitations. In contrast, association mapping fulfills linkage mapping and covers whole genome sequencing [153]. Whole-genome association studies or genome-wide association studies (GWAS) are becoming a standard tool for rapidly uncovering marker-trait associations in crops such as maize, where the generation of high-density markers is feasible and affordable. With the improvement in quality protein and yield of maize continuously for decades, the digestibility of stock has decreased drastically, ultimately making maize unsuitable for fodder. It is either due to the loss of alleles for digestible cell walls during breeding for stalk standability or genetic drift during breeding for grain yield [154]. Some breeders have attempted to improve maize’s fodder quality in the recent past, and genes or alleles have been identified for the same. Mutant genes, for example, the BMR, *wx*, Leafy1 (*Lfy1*), and *floury-2* genes, have been used to develop silage hybrids [155]. The brown-midrib 3 mutant (bm3) in maize (*Z. mays* L.), controlled by the caffeic acid O-methyl transferase (COMT) locus, has a positive influence on maize fodder quality [156]. 

Zein and a group of researchers [154] evaluated European maize lines for the nucleotide diversity and linkage disequilibrium (LD) pattern across 2.3 kb of the caffeic acid O-methyl transferase (COMT) locus. Andersen and coworkers [157] cloned and sequenced a Phenylalanine Ammonia-Lyase (PAL) genomic sequence from 32 maize inbred lines employed in forage maize breeding programs in Europe. Vinayan and a group of researchers [12] identified candidate genomic regions for fodder quality in testcross progenies of tropical origin using GBS and 55K Infinium chip data sets, rendering GBS the preferred technology to explain the phenotypic variance of complex traits. A total of 196 SPNs associated with acid (75), neutral detergent fiber (41), and in vitro dry matter digestibility (83) were identified by Wang and coworkers [158] in the mature stalk of maize, swaying its forage quality. Zhao and a group of researchers [159,160] studied the genetic influence of cadmium (Cd) and lead (Pb) accumulation in grains and leaves, utilizing GWAS and QTL mapping approaches to develop low-Pb-accumulating maize cultivars. Vinayan and coworkers [161] identified the genomic regions for fodder traits and conducted a genomic prediction study using 276 elite lines and 1026 DH lines from bi-parental crosses as prediction sets. A set of 955,690 SNPs were generated through GBS v2.7. 

**Figure 4:**
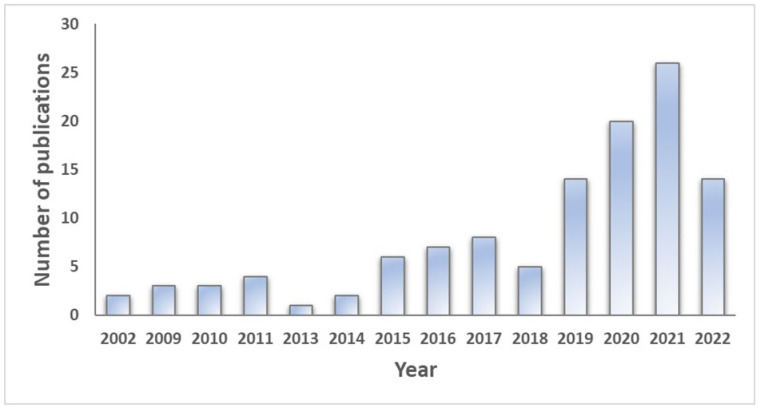
Number of published articles on transcriptomics in maize from 2002 to 2022. A web-search query through Google Scholar was used for a meta-analysis of 187 relevant articles. A keyword search was conducted in the abstract of the transcriptomics studies. Source: Google Scholar, dated 5 May 2022.

Various studies have provided insight into the transcript’s composition responsible for a differential gene expressed in specific cells or/and under specific cell treatments, causing phenotypic variation in maize growth, architecture, yield component, environmental response, pest and disease tolerance, and quality traits. A meta-analysis of 187 published articles from 2002 to 2022 revealed a consistent increase in the number of studies on transcriptomics in maize, though this has become significant in the past four years (Figure 4). These studies are primarily focused on the use of transcriptomics to identify tolerance/resistance to biotic and abiotic stresses in maize based on keyword search. A transcriptome study of maize root hair revealed that 3% of all genes were expressed in the root, comparable to the 4% found in Arabidopsis. Most of these genes are functionally related to energy metabolism, suggesting a high energy requirement for rapid cell division and root hair functioning [162]. Liu and Zhang’s [163] study identified six genes from transcriptome comparisons and correlation signaling network analysis involved in the regulation of the HY5 module and the MAPK cascade in the presence of blue light, controlling the stomata distribution and development in maize. Transcriptome analysis and qRT-PCR validation experiments of maize roots infected with *Holotrichia parallela* larvae established the expression of 12 differentially expressed genes associated with jasmonic acid mediated signaling and benzoxazinoid biosynthesis pathways responsible for root defense mechanisms against attack in maize [164]. Zhou et al. [165] investigated the mechanism of drought stress tolerance in maize using bulked segregant transcriptome analysis (BSTA). They revealed that alternative splicing, transcription regulation, and hormone metabolism are common mechanisms for maize’s response to drought stress. In a similar study, Du et al. [166] found *GRMZM2G055704* as a candidate gene controlling waterlogging tolerance in maize from BSTA, qRT-PCR validation, and QTL association studies. Transcriptome profiling of distinct maize inbred lines led to the discovery of highly expressed four candidate genes on chromosome 2, conferring resistance to Gibberella ear rot disease in maize [167]. From some of these results, it is evident that transcriptomics in maize allows for the large-scale identification of critical regulatory elements for tolerance to biotic and abiotic stresses [168,169,170], gene function annotation [171,172,173], and candidate gene identification [25,170,174]. This information will provide breeders with much genetic information for developing improved maize varieties considering the prevailing and anticipated economic, ecological, and environmental challenges to ensure food security.

The field of proteomics has garnered the attention of many scientists to analyze the differences in physiological conditions at proteomic levels under different stresses. For instance, to assess the changes at proteome levels in the case of corn infected by the Asian corn borer (*Ostrinia furnacalis*), Zhang et al. [175] conducted the proteomics of maize leaves and observed the presence of 62 defense-responsive proteins, especially pathogenesis-related protein 1 (PR1) and thioredoxin M-type, chloroplastic precursor, which demonstrated significant impact on the growth of larvae and pupae of the corn borer. Yet, in another study, using Isobaric Tag for Relative and Absolute Quantitation (iTRAQ) sets, Wang et al. [176] conducted comparative proteomic profiling of both susceptible and resistant lines against southern corn rust (*Puccinia polysora*) to find out that one specific remorin protein (ZmREM 1.3) is responsible for impeding resistance in resistant lines, information crucial for future breeding programs. Similarly, differentially expressed proteins have also been observed to protect corn against abiotic stress. For instance, drought stress during the grain filling period drastically reduces the productivity of crops. Hence, in an attempt to tease apart the role of defensive proteins in drought-tolerant varieties, Dong et al. [177] ran the comparative proteomic profiling of both drought-tolerant (ND476) and susceptible (ZX978) lines. They observed that 1655 defense-associated proteins (DAPs) are produced with the downregulation of redundant proteins to help plants save energy and fight stress.

The study of metabolomes helps to understand the response of maize plants under different stress conditions such as soil salinity, heat, drought, etc. [136]. A metabolomic study of salt-sensitive (PH4CV) and salt-tolerant (PH6WC) varieties of maize shows the difference in the accumulation of metabolites in roots and different metabolisms in seedlings. Under 100 mM NaCl conditions, glucose metabolism is induced in seedlings of the PH4CV cultivar, whereas significant acid metabolism was induced in seedlings of PH6WC. From the roots, 79 compounds were identified in the salt-sensitive cultivar and 85 were identified in the salt-tolerant cultivar, out of which 30 compounds were common in both cultivars and were associated with the basic metabolism of L-pyroglutamic acid, deoxyadenosine, adenine, cis-9-palmitoleic acid, and galactinol compounds. This knowledge helps us to understand the response of maize seedlings to salt stress [178]. Metabolic pathway analysis also reveals the effect of heat stress on the male sterility of pollen at the most susceptible tetrad stage in maize. The results show a reduction in pyruvate and an increase in sucrose components, whereas various genes associated with auxin production, signaling, and unfolded protein stresses remain unchanged. Genes related to heat stress; metabolic transcriptional regulation pathway altered even though at optimum conditions at which pollen can germinate. This leads to the conclusion that at the tetrad stage of pollen, short heat stress affects the basic metabolic pathways, leading to sterile pollen [179]. A study performed by Ganie et al. [136] in 2015 identified the metabolic pathways under phosphorus stress conditions, which will help develop approaches for increasing phosphorus efficiency. The metabolites were identified using gas chromatography-mass spectroscopy. Under P-limitation, sugar alcohols such as glucitol and mannitol increase, whereas fatty acids such as stigmasterol and cholesterol decrease, which are part of membrane fluidity. In the case of P starvation, plants scavenge the P from these fatty acids and thus disturb the membrane fluidity. It was also shown that the level of serine and glycine increased, which means the rate of photorespiration is also increased [136]. Using the nuclear resonance metabolome technique, the plasticity of leaves in maize plants was studied in response to heat and cold stress. The plastic response of maize plants under heat stress was different than that under cold stress in amino acid derivatives, biomass allocation, and other non-polar metabolites. It was also shown that the metabolic responsiveness in maize lines due to temperature differences was high. In contrast, the functional traits of maize show low plastic responsiveness, which indicates that metabolic and functional plasticity may play different roles in the adaptation of the maize plant to differences in temperature [180]. In another study, metabolic profiles of maize plants through combined effects of different stresses such as salinity, drought, and heat show that the metabolic profile of drought-stressed plants is more like salt-stressed plants than heat-stressed plants. Moreover, it was also found that the metabolic profile of drought-stressed plants, when combined with heat or salt stress, shows a different metabolic profile than either of the individual stresses. This shows the metabolic plasticity in maize to adapt to other environmental conditions. Thus, understanding the metabolic pathways under multi-stress conditions helps further optimize crop breeding for high-yielding plant varieties in changing climatic conditions [135].

The goal of maize forage breeding mainly revolves around yield and its stability, biotic and abiotic stress resistance, wider adaptability with photo and thermo insensitivity, and higher biomass. Due to global dominance, maize is grown under different environmental conditions, which require advanced phenotyping approaches to capture the unexploited variation. With the rise in global temperature, abiotic stresses such as drought are causing a severe threat to maize production. A further hindrance is caused due to high cost, low throughput, and labor-intensive conventional phenotyping. Plant height is usually measured at the end of crop growth and is directly correlated with yield. Adak et al. [181] used the weekly temporal flights and showed that variation captured in plant height at earlier stages is more predominant in predicting yield, and various QTLs are associated with plant height, which was previously assumed to be an oligemic trait. Plant stress phenotyping, especially for biotic and abiotic stress, was recently accomplished using close-range hyperspectral sensors as a promising non-invasive tool. These sensors monitor the physiological and biochemical changes occurring in the plants during different stresses based on the water content, plant organs, photosynthetic apparatus, and internal and external leaf structure. Wu and coworkers [134] performed an extensive study using 368 maize genotypes, where multiple optical images were collected with hyperspectral sensors, color and X-ray computed tomography images over 98 days under drought and controlled conditions. With these vast phenotypic datasets, the authors identified 1529 and 2318 significant QTLs and candidate genes showing drought-tolerant responses. Although various developments have occurred for above-ground phenotyping using proximal and aerial platforms in maize, little is known about the below-ground traits. The complexity of roots can be interfered with by several factors, such as their role in water and nitrogen efficiency, response to biotic/abiotic stresses, and overall plant health.

### 1.4. Oats

Oat (*Avena sativa* L.) is a nutritionally important cereal crop produced for food, feed, and forage [182,183]. It contains minerals, proteins, fiber, vitamins, lipids, unsaturated fatty acids, as well as other biochemical compounds that play a role in preventing diseases such as colon cancer, type II diabetes, cardiovascular disorders, etc. [184]. Cultivated oat (*Avena sativa* L.) is an allohexaploid (2*n* = 6x = 42; 1C-value = 13.2 (pg) DNA) with three diploid sub-genomes (AA, CC, DD). The oat grain has the highest protein content among cereals, which is approx. 12 to 20%, and lower fat content (<8%) in the groat [185]. Oat is the sixth-ranked cereal crop and has received significant attention for its positive and consistent health benefits and livestock feed [186,187]. It has the benefits of lowering blood cholesterol and reducing the risk of cardiovascular diseases [188] due to the presence of high soluble fiber (β-glucan) and antioxidants such as tocopherol and tocotrienol [189]. The large production is due to the adaptation of this crop to various soil types, where oats perform better than the other small grain cereals, and metabolomics has proven pivotal in studying the adaptive responses of plants to various abiotic and biotic stresses (plant × environment interactions) [190].

Oat genomic research has lagged as compared to the other major crops, such as rice and maize, due to the large size and complexity of its genome, lack of sequence data and sequence redundancy among sub-genomes, numerous chromosomal rearrangements, and chromosome-deficient cytogenetic stocks [191,192]. The genetic research in oat was initiated with the development of the first RFLP map developed by O’Donoughue and co-workers [193] in diploid oat. Different DNA markers such as diversity array technology (DArT) and single nucleotide polymorphism (SNPs) have been used [194] to develop genetic maps using different populations such as ‘Kanota × Ogle (K × O)’ [193], ‘Ogle1040 × TAM O-301 (O × T)’ [195], ‘Terra × Marion’ [196] and ‘Ogle × MAM17-5 (O × M)’ [197]. The first physical anchored consensus hexaploid oat map based on the previous six populations was developed by combining 985 SNPs and 68 previously published markers and has 21 linkage groups with a total length of 1838 cM [198]. Chaffin and a group of researchers [199] constructed the consensus map for hexaploid oat using cDNA-derived SNPs and GBS from 12 recombinant inbred lines population developed using 19 parents. This consensus map consists of 7202 markers with a total map length of 2843 cM covering 21 consensus chromosomes, which will accelerate the oat genomic research and provide a better understanding of the organization of the oat genome. Recently, in 2021, Pepsico and Corteva Agriscience released the whole genome sequence of hexaploid oat line OT3098, which can be accessed at https://wheat.pw.usda.gov/jb/?data=/ggds/oat-ot3098-pepsico (accessed on 5 May 2022). Advancements in marker technology, along with the development of new maps and integration of maps from multiple populations into a single consensus map, can accelerate oat’s genetic and genomic research [200]. These maps have been widely explored in various oat genetic analyses to identify minor quantitative trait loci (QTLs) associated with multiple agronomic traits using experimental bi-parental populations and a diverse panel of oat accessions. In recent years, genome-wide association studies (GWAS) have been reported, primarily focused on biotic stress such as crown rust and quality traits such as β-glucan concentrations [201,202]. Oat crown rust, caused by *Puccinia coronata* f. sp. *avenae*, is a major constraint to oat production worldwide, causing a significant reduction in grain yield, forage, and seed quality [203,204]. Genetic resistance is an effective and economical method of controlling crown rust in oats. Major race-specific resistance genes for crown rust (Pc) have been identified in oat germplasm [205], such as Pc38 on linkage group Mrg02 (chromosome 9D; [206], Pc48 on Mrg20 [206], Pc58a on Mrg02 [207,208], Pc68 on Mrg19 [209], Pc71 on Mrg21 [210], Pc91 on the translocated chromosome 7C-17A [211], and PcKM on Mrg08 [212], but they provided durable resistance only for a short period [213,214]. Alternative strategies such as partial/adult plant resistance can be used to control oat crown rust effectively. Chong [215] identified two genes controlling adult plant crown rust resistance using 157 F7:9 recombinant inbred lines (RILs) developed from an AC Assiniboia × MN841801 cross. Portyanko and co-workers [216] identified four major and three minor QTLs associated with adult plant resistance in a mapping population of MN841801-1 × Noble-2 cross. Acevedo and co-workers [217] also studied crown rust resistance in 150 F6:9 MN841801-1 × Noble-2 RILs and found eight QTLs associated with MN841801-1 alleles. In these studies, adult-plant rust resistance QTLs have been mapped on linkage groups Mrg02, Mrg06, Mrg08, Mrg12, Mrg17, and Mrg20 [216,217,218,219,220]. Association mapping for crown rust resistance has identified QTLs on linkage groups Mrg01, Mrg03, Mrg08, Mrg20, Mrg23, and Mrg28 [221,222] using the Diversity Arrays Technology. Klos and a group of researchers [223] conducted the first multi-environment genome-wide association mapping of crown rust resistance at seedling and adult plant stages using 2972 SNPs genotyped on 631 elite oat lines under both controlled and field conditions. They found 29 SNPs on 12 linkage groups associated with crown rust reaction in at least one experiment. This study identified the QTLs in the genomic regions carrying seedling resistance genes such as *Pc48*, *Pc58a*, *Pc68*, *Pc71*, *Pc91*, and *PcKM*.

Powdery mildew caused by *Blumeria graminis* sp. *avenae* is another primary disease that affects oat yield, and various studies have reported the presence of several genes in wild oat species [224]. Simons and co-workers [225] identified four major genes, *Eg-1*, *Eg-2*, *Eg-3*, and *Eg-4*, for powdery mildew. Yu and Herrmann [224] introgressed the powdery mildew resistance gene *Eg-5* from *Avena macrostachya* into cultivated hexaploid oat and mapped this gene on linkage group 22 of the Kanota × Ogle mapping population map [226]. Montilla-Bascon and a group of researchers [222] also conducted association mapping for powdery mildew by using 177 oat accessions genotyped using 31 simple sequence repeats and 1500 DArT markers and found one DArT sequence, oPT-5014, associated with powdery mildew results at the adult plant stage. Oat grain has high nutritional value due to protein, fiber (β-glucan), and oil. Oat grain has <8% oil content in the form of triacylglycerols (TAGs). Quality improvement is an essential objective for oat breeding programs. Kianian et al. [227] conducted the first QTL analysis for oil content using two different populations, the Kanota × Ogle mapping population (K × O) and the Kanota × Marion (K × M) mapping population. They found three QTLs for oil content in each population. In 2000, Kianian et al. [227] used these two populations, K × O and K × M, to study the inheritance of β-glucan and found seven and four QTLs associated with β-glucan in the K × O and K × M populations, respectively. De Koeyer et al. [196] identified six QTLs for oil content in the Terra × Marion mapping population, and Zhu and a group of researchers [197] also reported six QTLs in the Ogle × MAM17-5 (O × M) mapping population. Jackson et al. [189] used the Ogle1040 × TAM O-301 (OT) linkage map and mapping population. They found five, six, and one QTLs associated with α-tocotrienol, α-tocopherol, and total tocopherol concentrations, respectively. Tanhuanpää and coworkers [228] used the Aslak × Matilda (A × M) mapping population and found eight QTLs associated with oil content. Kianian and a group of researchers [227] proposed that acetyl coenzyme A carboxylase (ACCase) might be the potential candidate gene underlying the genomic regions associated with oil content variations in these populations. Eight QTLs were identified by Hizbai and a group of researchers [229] using the Dal × Exeter (D × E) mapping population associated with the oil content. Hizbai et al. [229] also found that the genomic regions associated with variations in different fatty acids (oleic acid, linoleic acid, linolenic acid, etc.) coincided with the oil content QTLs and may have a pleiotropic effect. GWAS for β-glucan was conducted by Asaro and coworkers [202] using 446 elite oat breeding lines genotyped by DArT markers, and 24 DarT markers were found to be associated with β-glucan. The genetic positions of 15 out of these 24 markers colocalized with QTLs reported in previous bi-parental mapping studies [196,201,230]. Based on sequence homology to rice, Newell and a group of researchers [201] found that one of the DArT markers sequences located on rice chromosome 7, which is adjacent to the *β-glucan synthese CslF* gene family, was found in GWAS. More studies are needed to fine-map identified QTLs and develop markers for marker-assisted selection to breed for high protein and fiber content in oats.

Various studies reported the synteny between oats chromosomes with the *Brachypodium distachyon* chromosome, the Oryza sativa chromosome, and wheat chromosomes controlling multiple disease resistance and quality traits. For example, synteny between the 4C oat chromosome and chromosome 4 of *Brachypodium distachyon*, chromosome 5BL of wheat, and chromosome 9 of rice carries the resistance genes for crown rust. Similarly, model grass *Brachypodium distachyon* has accelerated oat genomic research by assisting in rearranging the oat sub-genomes and differentiating the homologous relationships between oat linkage groups [231]. The synteny relationships among the genomes of different cereals accelerate the identification and annotation of genes in oats. In summary, the genomic regions associated with quality traits (protein and oil content) and resistance to biotic stresses (crown rust and powdery mildew) in different mapping populations/accessions will help to elucidate the genes and various metabolic pathways to improve the oat germplasm and transfer of favorable alleles that protect against biotic stress. Advancements in high genotyping technologies (DArT markers and GBS) for high-resolution mapping, new statistical approaches, and implications of genomic selection might be employed to predict the breeding values using marker information to speed the oat breeding process in a short period. 

The transcriptome analysis lagged in oats compared to other cereal crops due to the complexity of the genome and the challenges of differentiating the homologs and paralogs. RNA-seq is a powerful tool for transcriptome research, even for crops without reference genomes such as oat. Gutierrez-Gonzalez and a group of researchers [232] conducted the first comprehensive transcriptome study in oat seeds. They successfully studied the expression of genes involved in the biosynthetic pathways of avenanthramides, tocols, and beta-glucans. The resulting *dnOST* (*de novo* Oat Seed Transcriptome) transcript assembly has nearly 75-fold average coverage and will be a valuable tool for further transcriptomic research in oats. Jinqiu et al. [233] studied the transcriptomic expression in oats in response to altitude stress. They found 11,639 differentially expressed genes among low and high altitudes that might provide resistance to altitude stress. Recent advances in high-throughput genome sequencing and transcriptome profiling technologies will enable the quantification of gene expression for yield and quality-related traits in oats.

Therefore, since oats act as a phytoremediation crop, it is plausible to expect that there is some inbuilt adaptive mechanism to help plants combat different stressful conditions. Zhao and coworkers [37] observed the upregulation of 164 proteins with a subsequent downregulation of 241 proteins in shoots under alkali stress. Similarly, many resistance proteins such as GDSL esterase lipase in roots and late embryogenesis abundant (LEA) in shoots are accumulated under high pH and alkali stress. Further, escalating levels of glutathione and ascorbate play an essential role in plant defense against powdery mildew (*Blumeria grammis*) in oats [234]. 

In one study, it was found that in the Flega variety (susceptible to drought) of oats, there was a dramatic decrease in polar lipid in correlation with the increase of triglycerides (TGA) and free fatty acids (FFA) and in the resistant variety (Patones), there was a slight decrease in polar lipid in the correlation with increased diglycerides in response to drought stress. It was also found that in adverse environmental conditions, plants generally increase the metabolite biosynthesis to replenish the depleted molecule, which was also observed by Xu et al. [182] during the analysis of metabolites in salt-sensitive cultivar BY5 (Baiyan5) and salt-tolerant cultivar BY2 (Baiyan 5) using chromatographic-mass spectroscopy. The results showed the accumulation of more metabolites in BY2 than in By5 under salt stress. This metabolic pathway identification can aid biomarker selection breeding programs for developing salt-tolerant crops. So, the sugars accumulated in salt-tolerant cultivars were sucrose, isomaltose, and sophorose, and in susceptible cultivars were leucrose, trehalose, tagatose, and isomaltose. In salt tolerant genotypes, enrichment of sucrose is believed to be a plant defense strategy against salt stress. Similarly, accumulated amino acids reported were isoleucine, asparagine, serine, and glutamine in the salt-tolerant cultivar and proline, inosine, and asparagine in the susceptible cultivar. The enrichment of these metabolites helps oats improve the neutralization of excess reactive oxygen species and relieve salt stress. The metabolic profiling of oat seeds with Ultra High-Performance Liquid Chromatography-Mass Spectrometry (UHPLC-MS) helps us understand the effect of nitrogen application on seed quality with respect to the accumulation of primary and secondary metabolites. The total organic acid metabolism decreases, whereas the amino acid metabolism increases with nitrogen supplementation [235]. Metabolomic studies of various oat species, subspecies, and individual accessions can also be used as a biochemical fingerprint for identifying oat plants at individual accession levels based on biochemical characteristics. The metabolomic profiles of different oat species (*Avena strigosa* Schreb (diploid), *A. abyssinica* Hochst (tetraploid), and *A. sativa* L. (hexaploid)) give the informative indicators for separation of these species at different ploidy levels. Metabolites such as xylitol, undecylic, glutamic acid, isofucosterol, MAG-118:0, linolenic, methylmalonic, and undecylic acid were identified using an Agilent 6850 gas chromatograph and can be used as informative indicators [184]. A study performed by Pretorious and coworkers where used metabolomics for the identification of signature biochemical compounds that can be used for developing the differential metabolic profiles of various oat cultivars (Dunnart, Magnifico, Pallinup, SWK001, and Overberg). The metabolites were analyzed with ultra-high-performance liquid chromatography (UHPLC) coupled with a mass spectrometer analytical platform. Biomarker compounds among the respective cultivars were profiled into different classes, including amino acids, fatty acids, carboxylic acids, phenolic compounds like hydroxybenzoic and hydroxycinnamic acids, and flavonoids. Therefore, metabolomics provides us with great insight into understanding the biochemistry and physiology of crops, which will be helpful in overcoming the limitations of marker-assisted breeding programs for crop improvement [190].

Phenomics-assisted selection is becoming popular in oats. Some studies have been conducted on the different traits of interest till today using multispectral and hyperspectral cameras. Some crucial characteristics of oats are biomass, nutrients, seed/kernel, biotic-abiotic stresses, etc. [29]. So, we can predict the better future of oat breeding for fodder purposes using phenomics-assisted selection.

### 1.5. Alfalfa

Alfalfa (*Medicago sativa* L.) is the most essential perennial forage crop globally and is grown on more than 30 million hectares worldwide [236]. It is the fourth major cash crop in the United States, accounting for $9.7 billion in production (USDA-NASS, 2021) after corn, soybeans, and wheat (www.naaic.org, accessed on 12 June 2022) and in the world because of its highly nutritious forage and broad adaptability [237]. Cultivated alfalfa (*Medicago sativa* L.) is a perennial, autotetraploid (2*n* = 4x = 32), allogamous, and heterozygous species with a basic chromosome number of eight and a genome size of 800–1000 Mb [238]. Primary breeding goals in alfalfa include increasing yield, enhancing nutritive quality, and improving tolerance to biotic and abiotic factors that challenge alfalfa production. Alfalfa exhibits severe inbreeding depression, precluding the development of inbred lines [239].

Most of the early efforts in alfalfa breeding were focused on improving resistance to biotic stresses and forage nutritional quality and not on yield. Yields in alfalfa have been stagnant for the last few decades. Major QTL related to yield and morphological traits, including fall dormancy and winter-hardiness, persistence, viability, self-fertility, and resistance to various biotic and abiotic stresses, have been mapped in alfalfa, primarily in tetraploid populations [240]. A good review has already been published in alfalfa for progress in breeding using traditional breeding until 2018 [240,241]. Initially, the genetic dissection of essential traits, including forage quality and yield in tetraploid alfalfa, was difficult due to the complex segregation of alleles in tetrasomic inheritance and difficulties in analyzing polyploid linkage relationships [242]. Thus, early genetic linkage mapping [242,243,244] was conducted in diploid (2*n* = 2x = 16) species of alfalfa using molecular markers such as restriction fragment length polymorphism (RFLP), random amplified polymorphic DNA (RAPD), and SSR markers. Later, genetic mapping was extended to tetraploid alfalfa in backcross populations using single-dose allele analysis with RFLP and SSR markers, where the presence or absence of each marker allele was scored independently of the other alleles at the same locus [244,245,246,247]. Such alleles follow disomic inheritance, which could be mapped using diploid linkage mapping software. With the development of TetraploidMap [248] software, a linkage mapping tool and its updates thereafter for autotetraploid species, genetic mapping in tetraploid alfalfa was greatly enhanced. As DNA marker technology advanced, a saturated genetic linkage map of autotetraploid *M. sativa* was generated by Li et al. [249] using genotyping-by-sequencing (GBS) methods. That map contained 3561 SNP markers on 64 linkage groups across both parents, with an average density of one marker per 1.5 and 1.0 cM for the maternal and paternal haplotype maps, respectively. Research from multiple projects has demonstrated that the alfalfa linkage groups were found to be highly syntenic with the diploid *M. truncatula* model *Medicago* genome sequence reference [246,249,250,251,252].

In the last decade, several association mapping studies have been conducted using diploid or tetraploid alfalfa breeding populations to identify marker-trait associations for biomass yield and stem composition [253,254,255], fiber-related traits and digestibility [158], and CP concentrations [256] using SSR markers. Recent advances in next-generation sequencing have provided a new strategy to generate cost-effective, high-density, genome-wide single nucleotide polymorphism (SNP) sets [257]. Moreover, it is difficult and costly, to date, to achieve 60x coverage in genome sequencing or GBS approaches to account for tetraploid allele dosage and distinguish between three heterozygous classes to conduct GWAS and GS. Earlier studies combined the three heterozygous genotype classes to conduct a genomic study [237] or use a single-dose allele in a bi-parental population [258]. Zhang and coworkers [259] mapped quantitative trait loci (QTL) for flowering time in autotetraploid alfalfa (*Medicago sativa* L.) using SNP markers. With the development of new software such as GWASpoly [260], it is now possible to account for three different heterozygous classes using biallelic SNP markers and conduct genomic analyses using different polyploid gene action models. Now that the autotetraploid alfalfa genome has been sequenced [261], this resource can be used along with *M. truncatula* as reference genomes for GBS analysis in alfalfa. 

The application of genomic predictions to alfalfa biomass yield and forage quality breeding has been initiated recently [13,237,262,263]. Li et al. [262] used clonal ramets from 185 to 190 individuals from two selection cycles of a tetraploid alfalfa breeding population to conduct genomic predictions in a three-location study and reported prediction accuracies of 0.43 to 0.66 within each location. They also attempted to validate the models across two locations and reported 0.21 to 0.61 prediction accuracies. Jia et al. [264] used 322 individual genotypes from 75 diverse alfalfa populations to test three Bayesian genomic prediction models for 25 agronomic traits in alfalfa. These included 15 forage quality traits and ten other traits such as dry matter, plant regrowth, fall dormancy, leaf to stem ratio, etc. In their study, prediction accuracies ranged between 0.0021 and 0.6485, and no significant differences were observed between BayesA, BayesB, and BayesC models. Medina et al. [263] also conducted genomic prediction among 304 clonally propagated alfalfa plants for biomass yield under salt stress. They reported that prediction accuracies ranged from 0.087 to 0.457 in different harvests. Predicting the breeding value of candidate parent genotypes for synthetic variety development of outbred species, such as alfalfa, can also be pursued by genotyping a set of parent plants and phenotyping their half-sib progenies [236,237,240] genotyped parents and phenotyped half-sib progenies to test seven different parametric and machine learning genomic selection models for predicting biomass yield in two different alfalfa populations. The support vector machine (SVM) models performed best, with the highest prediction accuracies. Similarly, Biazzi et al. [13] used half-sib progenies of 152 genotypes for phenotyping forage quality traits in alfalfa and achieved prediction accuracies ranging from 0.1 to 0.4 for different traits for five other GS models. In this later study, rrBLUP, BayesA and B, and Bayesian LASSO tended to outperform the SVM model with a linear kernel. Efforts are being made to fully sequence cultivated alfalfa at the diploid level (CADL) and to develop the pangenome to explore core genes in alfalfa. Through comparing cultivar sequence information and using GWAS for traits such as nutritional quality and yield, forage crops can be improved. 

The Affymetrix GeneChip^®^ Medicago genome array was released in 2005 and included over 52,000 Medicago probe sets designed from 32,167 *M. truncatula* ESTs, 18,733 gene predictions from *M. truncatula* genome sequences, and 1896 cDNAs from M. sativa. This GeneChip^®^ was used for cross-species transcriptomic studies in alfalfa [265,266,267]. Lignin content in plants is a biochemically well characterized trait and directly affects the forage quality as a livestock feed. Therefore, cell wall composition, which mainly consists of cellulose and lignin, in stems was chosen for transcript profiling in earlier studies in alfalfa [267,268,269]. Later, several studies contributed to the transcriptome sequencing of alfalfa targeting different genes for salt tolerance [270,271,272], drought tolerance [273], freezing tolerance [274], and bacterial stem blight disease [275] traits using NGS technologies or RNA-Seq. In 2011, the first publicly available *Medicago sativa* gene index (MSGI 1.0) was developed by Yang et al. [268], using the elongating stem and post-elongation stem internodes from two alfalfa genotypes with differing cell wall composition in stems. The MGSI 1.0 has 124,025 unique sequences, including 22,729 tentative consensus sequences (TCs), 22,315 singletons, and 78,981 pseudo-singletons. A high-resolution melting [HRM] technology has also been implemented to obtain 54,216 unique sequences in a population generated using two diverse genotypes differing in water stress sensitivity [273]. Li and coworkers [269] conducted transcript profiling using Illumina short reads from stem tissues at different growth stages with 27 diverse alfalfa germplasm, including commercial and wild genotypes, and identified 604,164 SNPs and InDels in the alfalfa population. Large scale transcriptomic analysis was conducted by Liu et al. [276] using 15 types of different tissues, including germinating seeds, young leaves, young stems, mature stems, mature pods to callus cells from one alfalfa cultivar. This extensive RNA-Seq analysis generated 40,433 unigenes in addition to 1649 EST-SSRs markers. The MSGI 1.2 was assembled by adding transcripts from multiple plant tissues such as roots, nodules, leaves, flowers, and stem internodes at multiple growth stages, representing 112,626 unique genes using two diverse subspecies of *M. sativa* [277]. Shu and coworkers [274] reported that C-repeat binding factors (CBF) genes play an important role in freezing tolerance in alfalfa. Later in 2018, Luo and coworkers [278] used the PacBio Iso-Seq sequencing technology to generate the full-length transcripts from alfalfa roots under salt stress conditions in a single cultivar to identify 5011 and 4546 differentially expressed (DEG) genes with NaCl and mannitol after 24 h of treatment. Recently, Illumina short read transcripts and Iso-Seq long read transcripts were used for generating a pan-transcriptome to identify 1,124,275 unique isoforms and 91,378 genes in response to drought and salt stress using three diverse alfalfa germplasms for these two traits [279]. Jiang and coworkers [280] combined genomic and transcriptomic analysis in an F1 population developed using two parental genotypes with varying leaf sizes. They identified seven candidate genes associated with leaf development in five major QTL regions using association studies and 2443 leaf-specific genes and 3770 differentially expressed genes using RNA-Seq analysis. With the advancement in transcriptomic technologies, studies can be conducted to identify genes for various diseases using different genotypes from various plant tissue replicates at multiple stages, which is currently lacking in alfalfa.

Recent advances in metabolomics and proteomics technologies greatly expedite the identification and characterization of natural products and their associated metabolites. Proteomics and metabolomics can remarkably examine the balance between carbon and nitrogen metabolism under stress conditions in alfalfa during interactions with nitrogen-fixing bacteria [281,282]. Metabolomic analysis of alfalfa (*Medicago sativa* L.) root-symbiotic rhizobia responses under alkali stress. Water stress limits nitrogen fixation in nodules by reducing nitrogenase activity [282] and Rubisco availability in leaves [282]. Metabolomics has been utilized to elucidate the internal causes of nutrient change at different developmental stages in alfalfa plants [283]. Furthermore, a study found that a water deficit significantly enhanced the alfalfa’s freezing tolerance. This was correlated with increased soluble sugar, amino acid, and lipid and lipid-like molecule content using metabolomic analyses. This study improves our understanding of the relationships between metabolites and freezing tolerance following cold acclimation and freezing temperatures, suggesting that metabolites play essential roles in enhancing the freezing tolerance of alfalfa. 

Saline-alkali stress is the chief abiotic stress in alfalfa, significantly affecting the yield and quality of the crop. Several attempts have been made to understand the adaptive mechanisms against these stresses in resistant crops. One such study elucidated the induction of 226 differentially abundant proteins (DAP) under salt stress, leading to an elevation in glutathione, an antioxidant and oxidation-reduction pathway to adapt the plant to salt stress [10]. In another similar study, proteomic analysis between drought tolerant variety of alfalfa Longzhong and drought-sensitive variety named Gannong No.3 revealed the accumulation of 142 DAPs involved in stress, defense, transmembrane transport, and cytoskeleton metabolism to increase the osmotic adjustment capacity of the Longzhang variety [284]. Comparative proteomic analysis has helped tremendously in deciphering different types of proteins responsible for adaptive mechanisms in alfalfa.

Moreover, metabolomics can be utilized to evaluate the metabolic response of the root-nodule symbiosis in alfalfa under alkali stress [281]. Physiological analysis and metabolic profiling using GC-TOF/MS comparative analysis employed to identify metabolites and pathways that change after *Rhizobium* inoculation revealed that RI plants accumulated more antioxidants (SOD, POD, GSH), osmolytes (sugar, glycols, proline), organic acids (succinic acid, fumaric acid, and alpha-ketoglutaric acid), and metabolites that are involved in nitrogen fixation. This study revealed a distinct metabolic profile is induced in nodulized plants with putative alkali tolerance compared to non-nodulated alfalfa plants. The effects of *Bacillus subtilis* inoculation on the growth and Cd uptake of alfalfa were evaluated using metabolomics analyses [285]. The results indicated that inoculation significantly decreased the amount of plant malondialdehyde (MDA) and improved the activities of plant antioxidant enzymes and soil nutrient cycling-involved enzymes, thereby promoting biomass by 29.4%. Moreover, a study by [286] found the metabolic mechanisms underlying the response of alfalfa reproductive organs to boron deficiency and surplus, which could provide new strategies for improving seed yield and quality. Boron deficiency leads to the excessive accumulation of sugars in flowers and phenolic compounds in seeds, causing abscission of reproductive organs and then reduced yield and quality of seeds. Boron surplus caused a severe reduction in the metabolites associated with amino acid and carbohydrate metabolism, causing flowers to fall before seed set, thus reducing seed yield.

Phenotyping for biomass yield requires significant resources [287], which are time and labor-intensive. Some critical traits for alfalfa breeding are biomass, plant height, persistence, biotic-abiotic stress, root architecture, etc. Biomass [141,288,289,290] is the most important trait for alfalfa breeding. Remote sensing techniques have enabled efficient and non-destructive estimation of biomass in alfalfa [289], such as screening large breeding populations [291]. Breeding alfalfa for biomass involves repeated and numerous phenotyping efforts, which is laborious and costly [292]. Biswas and coworkers [292] found that phenomic-assisted selection can reduce up to 70% of manual data collection but still predict the biomass yield correctly, help select high-yielding alfalfa cultivars, and significantly contribute to breeding. According to Cazenave et al. [291], high throughput phenotyping (HTP) can identify minor differences in alfalfa yield when screening diverse germplasm. More recently, HTP enhanced the efficacy of the selection process for biomass in small plots in alfalfa breeding populations [293] and provided a good prediction of biomass in bigger plots [289]. Considering all that research, phenomics can lessen the challenge of biomass measurement in larger populations for alfalfa breeding programs aimed at improving biomass yield. Therefore, applying HTP or phenomics can simplify the phenotyping process for alfalfa biomass. To our knowledge, few studies [141,290,291,294,295] have been conducted on phenomics-assisted research for other traits such as root architecture [295], abiotic stress [291], nutritional value [296], seed identification or separation from other seeds [141,290,296]. However, it is becoming popular in other crops for reducing time and labor. Genomic selection (GS) is becoming a popular perennial crop breeding technique, requiring phenotyping data to make the prediction model predict yield or other traits. We found only one study [297] that used phenomic-assisted selection with GS to make the prediction model. Though some other research has been conducted on phenomic-assisted selection in alfalfa, still, there is a vast prospect of utilizing phenomic-assisted selection or high throughput phenotyping in alfalfa breeding. 

## 2. Conclusions and Future Prospect

There is no argument for the fact that the field of omics has grown by leaps and bounds, providing fast, robust, cutting-edge technology that enables fast and accurate detection of different problems in complex biological samples. However, many challenges still need to be conquered on the journey to produce smart, resilient, high-grain quality crops. Work has been conducted on the grain quality. However, the fodder traits still needed to be targeted in the crop improvement programs using techniques including genomics, transcriptomics, proteomics, metabolomics, and phenomics. Advances in genomic tools for breeding and developing improved cultivars for several crops have been made. However, there is scope for developing better performing fodder crop varieties that will be characterized by traits like stay-green, a higher number of tillers per plant, higher total biomass, good nutritional quality, and tolerance to biotic/abiotic stresses. 

The functional study is faster in crops such as Arabidopsis and rice, which have well-developed transformation systems. However, a high-efficiency and stable transgenic system for fodder crops is required to make the functional study less time-consuming. Transcriptomics techniques such as RNA sequencing and NGS have proved to be excellent tools for identifying genes/QTLs and constructing gene maps, which have expedited the process of crop improvement. There is still a considerable void in analyzing whole proteome levels of non-model species and agriculturally essential crops. Research is in its infancy stage in exploring the full potential of emerging technologies such as peptidomics, phosphoproteomics, and redox proteomics to dive deep into protein and molecular interactions. Phenomics brings genetics and physiology together and gives the possibility of studying the under-explored fields of plant science and revealing the molecular basis of several unmanageable plant activities. Breeding cultivars tolerant to stressed environments can be speeded by collecting, integrating, and utilizing phenomic data. However, technological advances for collecting, handling, and processing extensive data must be employed. Along with technological improvement, more robust and advanced bioinformatics tools need to be developed to interpret the large amounts of information gathered by all the above-mentioned omics. The advancement in omic technologies in terms of being eco-friendly, low-cost, and time-efficient will improve fodder crops.

## Data Availability

Not applicable.
